# Modern Imaging Guidelines for 3-D Modeling of Pediatric Solid Tumors: A New Era of Surgical Oncology Operative Planning

**DOI:** 10.3390/children12121622

**Published:** 2025-11-29

**Authors:** Zachary R. Abramson, Daniel J. Robertson, Nikhil R. Shah, Keyonna M. Williams, Mark L. Ryan, Lincoln M. Wong, Christopher Z. Lam, Osama Raslan, Eric Diaz, Joseph C. Fusco, Dhanashree Rajderkar, Alexander J. Towbin, Erika A. Newman, Andrew M. Davidoff, Erin G. Brown

**Affiliations:** 1 Department of Radiology, St. Jude Children’s Research Hospital, Memphis, TN 38120, USA; 2 Department of Surgery and Pediatrics, Children’s Hospital of Illinois, Peoria, IL 61637, USA; 3 Department of Surgery, CS Mott’s Children’s Hospital, Ann Arbor, MI 48109, USA; 4 Department of Surgery, Children’s Medical Center Dallas, Dallas, TX 75235, USA; 5 Department of Surgery, University of Texas Southwestern Medical Center, Dallas, TX 75390, USA; 6 Department of Radiology, Children’s Nebraska, University of Nebraska Medical Center, Omaha, NE 68198, USA; 7 Department of Diagnostic and Interventional Radiology, The Hospital for Sick Children, Toronto, ON M5G 1E8, Canada; 8 Department of Medical Imaging, University of Toronto, Toronto, ON M5S 1A1, Canada; 9 Department of Radiology, University of California Davis Medical Center, Sacramento, CA 95817, USA; 10 Department of Surgery, Monroe Carell Jr Children’s Hospital at Vanderbilt, Nashville, TN 37232, USA; joseph.fusco@vumc.org; 11 Department of Radiology, University of Florida, Gainesville, FL 32611, USA; 12 Department of Radiology, Cincinnati Children’s Hospital, Cincinnati, OH 45229, USA; 13 Department of Radiology, University of Cincinnati College of Medicine, Cincinnati, OH 45267, USA; 14 Department of Surgery, St. Jude Children’s Research Hospital, Memphis, TN 38120, USA; 15 Department of Surgery, University of California Davis Medical Center, Sacramento, CA 95817, USA; egbrown@ucdavis.edu

**Keywords:** pediatric cancer, pediatric surgical oncology, 3-D, model, imaging, operative planning

## Abstract

**Highlights:**

**What are the main findings?**

- Not all routine images obtained exhibit sufficient quality and guidelines for image-based 3D model creation and quality assurance are notably absent.
- Understanding the fundamental tenets of image quality (spatial resolution, contrast, noise) gives rise to the recommendations and guidelines developed here.

**What is the implication of the main finding?**

- When imaging is performed for the purpose of 3D modeling, image quality may need to be optimized above routine parameters and thin-section images must be archived.
- Even in the case of suboptimal imaging, 3D models can be created to facilitate surgical planning and patient/family education.

**Abstract:**

**Background**: Three-dimensional (3-D) models are gaining interest for surgical planning in the field of pediatric surgical oncology. The diverse tumor types in pediatric patients and the non-uniform presentation and anatomy make 3D modeling in pediatric oncology particularly challenging. In addition, centers have variability in technique and experience with these approaches. Guidelines for model creation and quality assurance are notably absent. **Objective:** We developed national recommendations pertaining to image acquisition and model creation for 3-D renderings based on the existing literature and multidisciplinary expertise. **Materials and Methods**: The literature was critically appraised, and the authors developed minimum standard guidelines for imaging and modeling pediatric solid tumors. Strength of recommendation scores for each guideline were independently determined by the authors. **Results:** Twelve specific guidelines were developed and scored with overall strength of recommendation ranging from moderate to very strong. Guidelines focused on image acquisition were uniformly scored very strongly while scores for those pertaining to segmentation and model display were slightly less consistent. **Conclusions**: We propose minimum standards for image acquisition and modern 3-D modeling of pediatric solid tumors based on literature and expertise. The overall level of agreement among our multidisciplinary team was high.

## 1. Introduction

Surgeons have traditionally relied on standard cross-sectional imaging for operative planning. Recent advancements utilize post-processing techniques to create virtual three-dimensional (3-D) models from existing 2-D computed tomography (CT) and magnetic resonance (MR) images. The addition of the third dimension allows for enhanced visualization of the spatial relationships of anatomic structures in relation to solid tumors as well as determination of tumor volumes in a manner not feasible with 2-D imaging. The meticulous examination of such structures in relation to surgical landmarks has improved the identification of anatomy crucial for surgical planning which, in turn, may improve surgical outcomes [1,2,3,4,5,6,7]. Three-dimensional models have already revolutionized maxillofacial and orthopedic surgery due to the ease of bone segmentation and have recently extended into pediatric fields for applications in congenital heart disease [6,8,9]. These tools have demonstrated clinical value for pre-operative decision-making, training, and education, and increasingly continue to gain traction in the surgical realm.

Pediatric surgical oncology is uniquely poised to benefit from this novel technology. The depth and breadth of this surgical subspecialty encompass a myriad of tumor types and anatomic sites. Additionally, the smaller anatomical structures in children as well as considerations for minimizing radiation exposure add complexity to adequately imaging these patients. The integration of 3-D technologies for pediatric solid tumors may improve tumor visualization, facilitate more accurate staging, and optimize pre-operative planning, thus inciting widespread interest in incorporating this into routine peri-operative practice [10,11,12]. To date, this technology has not yet been standardized within clinical practice and guidelines for image creation and quality assurance are absent. We, therefore, established a multidisciplinary team of pediatric surgeons, radiologists, and engineers with interest and experience utilizing 3-D technologies to develop guidelines for 3-D visualization within pediatric surgical oncology. The proposed guidelines will establish a minimum standard for both image acquisition and model creation based on the existing literature and institutional expertise.

## 2. Materials and Methods

The authors critically appraised the 3-D image modeling literature to make evidence-based recommendations related to the technique’s use for pediatric oncologic surgical planning. The quality of the literature was assessed using the Grading of Recommendations, Assessment, Development, and Evaluation (GRADE) system [13,14]. GRADE A is considered high-quality evidence while GRADE D is considered very low quality. After appraising the literature relevant to recommendations, a strength of recommendation (SOR) was provided by each author for each recommendation.

The SOR was determined through a survey of the working group members using an established methodology [13,15]. Each working group member was asked to independently score each recommendation on a scale of 1–4, with 1 representing a very strong recommendation and 4 representing a weak recommendation. An overall SOR score for each recommendation was calculated as the arithmetic mean of the individual scores. The overall SOR score was then converted into an ordinal scale as follows: 1–1.49, very strong recommendation; 1.5–1.99, strong recommendation; 2–2.99, moderate recommendation; and 3–4, weak recommendation.

## 3. Results

Twelve authors independently scored 12 recommendations, which are separated into guidelines pertaining to image acquisition (Table 1) and image post-processing (Table 2). The quality of the literature was assessed as Grade D, very low quality, due to a predominance of expert opinion and single institution studies. The guidelines and rationale are discussed below.

### 3.1. Image Acquisition

The ideal imaging study for 3-D modeling exhibits high spatial and contrast resolution with minimal noise. However, both scanner- and patient-dependent factors may limit image quality. The spatial resolution of modern CT scanners is limited by detector size. Meanwhile, the small size of pediatric patients and the need to limit radiation exposure when performing CT impose inherent limitations on the fidelity of images that can be obtained. Nevertheless, understanding the fundamental tenets of image quality provides a framework for creating imaging standards and guidelines for 3-D modeling.

### 3.2. Spatial Resolution

**Figure 1:**
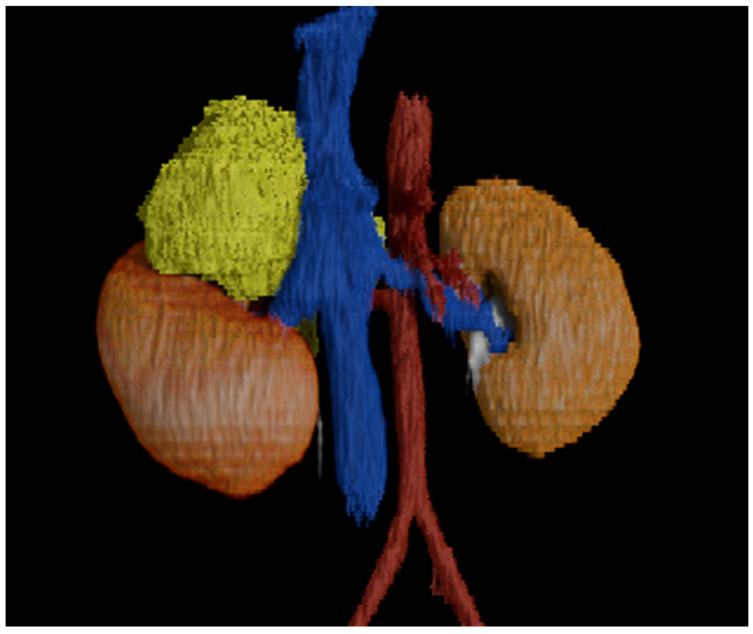
Three-dimensional CT-based rendering of a 7-year-old boy with right adrenal neuroblastoma (yellow). The stair-step effect is a result of using thicker sections for modeling.

Spatial resolution in CT can be defined as the number of line pairs on a phantom distinguishable per millimeter. Practically speaking, resolution on CT is limited by detector size, with most scanner detectors falling in the range of 0.5 to 0.625 mm. While the image data is acquired in thin sections matching the size of the detector, the data is often reconstructed in thicker sections on the scanner before being sent to the picture archives and communication system (PACS). The thicker sections improve signal-to-noise, reduce the number of images, and minimize data storage costs [16,17]. However, 3-D models created using thicker sections cause step-artifacts and could limit the ability to distinguish between adjacent small structures (Figure 1). In some institutions the thin image data is only stored on the scanner hardware for a short period of time before being permanently deleted.

**Figure 2:**
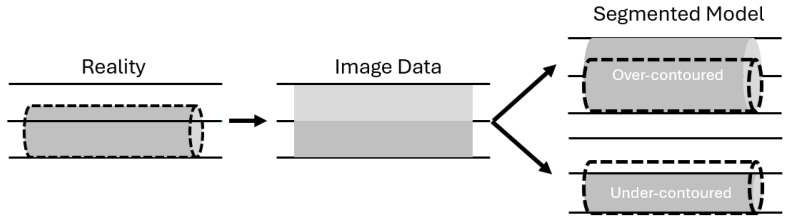
Volume averaging occurs when a structure partially occupies a slice. The partially occupied slice will exhibit a weighted average density of the structure of interest and adjacent tissue, resulting in a density that is typically less than that of the structure desired to be modeled. Automated and manual segmentation techniques may then incorporate or not incorporate the slice into the model, resulting in over- or under-contouring of the structure.

Thicker sections pose difficulty in modeling small structures due to an effect known as volume averaging, occurring when a structure of interest partially occupies a slice, resulting in a signal value representing an average of the structure itself and the surrounding tissues. This phenomenon increases in severity when using thicker slices. When modeling, the inclusion or exclusion of this averaged slice will lead to over- or under-contouring, respectively (Figure 2).

The other key component to resolution is voxel dimension. For 3-D modeling to be performed, voxels should ideally be isotropic, meaning that the x, y, and z dimensions are equivalent. Isotropic voxels allow a 3-D model to be viewed in any plane, including off-axis planes, without distortion and with the same resolution in all directions. ***Consequently, imaging obtained for the purposes of 3-D modeling should be as thin as possible with isotropic voxels (Evidence, 1.33 very strong)*** (Table 1).

Slice thickness on MRI, unlike CT, is not hardware dependent. Rather, the slice thickness achieved depends on the parameters set for a particular sequence. Higher resolution on MRI comes at the cost of increased noise and increased scan time, which can lead to artifacts and motion if not performed under anesthesia (Table 3). Additionally, many MR sequences may contain gaps between acquired slices, leading to gaps in modeling. In light of these technical limitations and considerations, ***MR sequences obtained for the purposes of 3-D modeling should be as thin as possible without gaps between slices (Evidence, 1.17 very strong)*** (Table 1).

### 3.3. Contrast Resolution

Contrast resolution refers to the difference in tissue density on CT or signal intensity on MRI between the tissue of interest and adjacent areas. On CT, the intrinsic contrast of soft-tissue structures is limited, necessitating the use of intravenous iodinated contrast material. Distinct from CT, MRI can generate excellent contrast resolution without the use of intravenous contrast material. Thus, the need for intravenous contrast material with MRI is dependent on the task to be performed and the structure(s) being evaluated. The most common MRI sequences applicable to 3-D modeling of soft-tissue tumors include multiplanar unenhanced T2-weighted fat-suppressed images and post-contrast T1-weighted fat-suppressed images.

When using CT, modeling of the abdomen necessitates visualization of structures that enhance with contrast in various phases (arterial, portal venous, systemic venous, excretory). Depending upon the structures needing visualization, a single-phase study may suffice. For example, renal veins and suprarenal IVC opacify shortly after or during the arterial phase, whereas the infrarenal IVC opacifies later. ***Determining the structures of interest, surrounding organs of interest, and their locations prior to the protocoling of the exam is important, as this may dictate the timing of contrast administration (Evidence, 1.25 very strong) (Table 1).*** However, visualization of structures which opacify at distinct times post-injection is often required. The most common way this is achieved is by multi-phase CT, which includes a single injection of contrast followed by serial imaging during the arterial, venous, and delayed phases. The resultant scans are then registered to one another, though interval motion and timing of phases during respiration sometimes impede accurate registration. The venous phases vary depending on the structures being imaged. Within the abdomen, renal, portal, and other visceral veins opacify at different times. A delayed phase may be used to image additional venous structures or the ureters, which are frequently encased by neuroblastoma. The more acquisitions used, however, the greater the radiation dose and potential for misregistration error.

**Figure 3:**
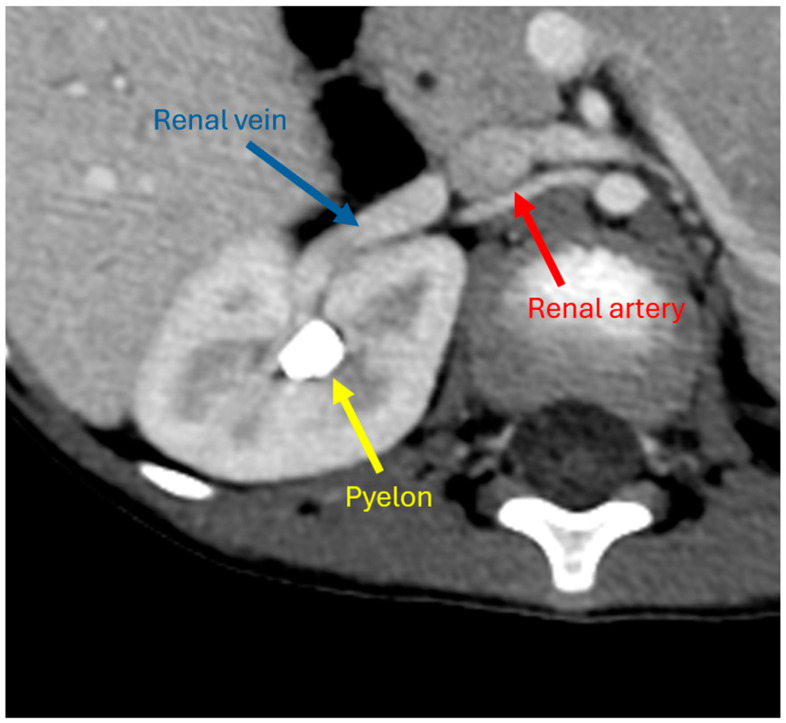
Axial contrast-enhanced CT of a 4-year-old girl with bilateral Wilms tumor obtained using a split-injection technique, achieving simultaneous opacification of the right renal artery (red), vein (blue), and ureter (yellow).

Alternatively, a split-injection technique can be utilized, where multiple contrast injections are separated in time, such that a single image acquisition is performed when the contrast boluses are in the desired vascular phases (Figure 3). This approach reduces overall radiation dose and obviates the need to register multiple phases but is more technique-sensitive and may require a higher dosage of contrast [18]. However, the simultaneous opacification of arteries and veins can make threshold-based segmentation difficult, particularly if the arteries and veins opacify to a similar degree. Depending on the desired structures to be modeled, a single-phase, multi-phase, or split-injection technique may be utilized. ***When both vascular and ureteral opacification is desired, a multi-phase or split-injection technique should be used (Evidence, 1.42 very strong)*** (Table 1).

**Figure 4:**
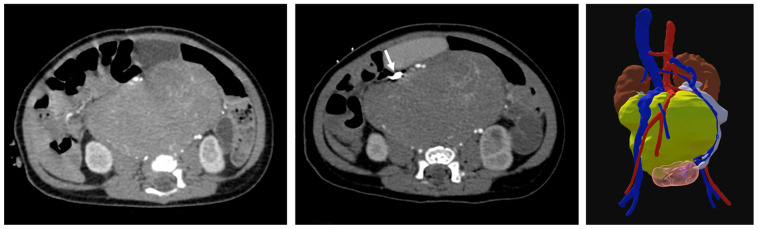
A 71-day-old boy with neuroblastoma centered at the aortic bifurcation. Initial pre-operative CT was obtained using an upper-extremity injection technique (**left**). The infrarenal IVC and iliac veins were not visualized. The exam was then repeated using a lower-extremity injection technique with excellent visualization of the IVC and extremity veins ((**middle**) arrow). Three-dimensional models were subsequently generated from the second scan for the purposes of surgical planning (**right**). Tumor shown in yellow, kidneys in orange, urinary bladder in pink, ureters in white, arteries in red, and veins in blue.

More nuanced discussion of timing contrast is needed when categorizing venous opacification as suprarenal IVC, infrarenal IVC, and hepatic. In children, particularly with good renal function, the renal veins and therefore the suprarenal IVC opacifies early, typically when the arteries are still well-opacified. The infrarenal IVC opacifies later from the lower extremities and the contrast is typically less dense due to dilution. Consequently, lower extremity venous injection is considered in cases where infrarenal IVC modeling is needed (Figure 4, Table 4).

Similarly to the suprarenal vs. infrarenal IVC, hepatic veins can be difficult to model due to suboptimal timing and/or dilution effect. Modeling of these veins can be critical, particularly in pediatric liver tumors where involvement of these structures impact resectability as well as chemotherapy [19]. A split injection can help by allowing greater time for hepatic vein opacification while still maintaining good arterial opacification. For larger patients, a slow injection may allow enough time for hepatic vein opacification. Imaging at the end of injection without delay often provides sufficient arterial opacification.

MR vascular imaging can be performed with and/or without contrast with high resolution. However, respiratory motion may hinder the ability to model small vessels, particularly accessory renal arteries, the artery of Adamkiewicz, and narrowed renal veins which may be encased by tumor. Nevertheless, ***MR-based 3-D modeling of vascular structures remains a viable alternative to CT, depending on patient-specific factors (Evidence, 1.83 strong)*** (Table 1). While MR liver studies with Gadoxetate are often used to evaluate liver tumors on account of its biliary excretion, traditional Gadobutrol agents may be superior for modeling due to improved T1 shortening and the fact that Gadoxestate has been shown to cause tachypnea, which degrades image quality of the hepatic veins near the diaphragm [20].

### 3.4. Noise

Noise refers to the random variation in the image within a homogeneous region of interest. There are many sources of image noise. When imaging with CT, there is an inverse correlation between image noise and radiation dose. The lower the radiation dose, the greater the image noise. The noise index is a parameter which can be adjusted at the scanner to control the amount of noise in the image. This is accomplished by modulating the current based on the attenuation profile of the scout radiograph. This modulation will alter the radiation dose received by the patient. Once acquired, image noise can be measured on the images using the signal-to-noise ratio, comparing the mean signal within the structure of interest to the standard deviation. ***When acquiring images for 3D modeling, a low noise index should be used, though this will vary based on specific scanner hardware and software capabilities (Evidence, 1.58 strong)***. There are a variety of post-processing reconstruction algorithms used to reduce image noise, including soft-kernel, iterative, and deep learning reconstruction techniques. These are often hardware- and software-specific and rapidly evolving. Therefore, no particular recommendation is made regarding the use of these techniques.

### 3.5. Image Post-Processing

#### 3.5.1. Segmentation

Segmentation involves the assignment of voxels within an image to a structure based upon the CT attenuation values or MR signal characteristics and relationship to other similar voxels. For instance, the attenuation of the aorta and its major branches is often very similar yet distinct from adjacent tissues, so selecting the lumen of the aorta and arteries is straightforward. Accurate and rapid segmentation is dependent on high contrast resolution. When contrast resolution is high, structures of interest are sufficiently distinct from those of adjacent tissues. In these instances, a threshold or mask can be applied to the image data allowing for auto-segmentation using one of several software tools. In adults, intra-abdominal fat provides excellent contrast, outlining solid and hollow viscus organs. Most pediatric patients lack abundant intra-abdominal fat, limiting the use of this intrinsic contrast for segmentation purposes. In these circumstances, manual segmentation can be performed, though it is time-intensive, user-dependent, and introduces a subjective component to 3-D modeling. Algorithms to replace the manual component are currently being generated but remain in their infancy [21,22,23,24]. ***For this reason, all segmentations should be reviewed by a radiologist with pediatric imaging expertise (Evidence, 1.42 very strong) (Table 2)***.

Occasionally, due to limited contrast resolution of soft-tissue structures on CT, the margins of a tumor can be ill-defined. MRI can often provide excellent contrast resolution. For example, segmentation of chest wall tumors on CT is difficult due to the similar attenuation characteristics of tumor, peritumoral fluid, and chest wall musculature. In these cases, T2-weighted MRI and post-contrast T1-weighted fat-suppressed sequences are helpful in delineating tumor margins. Similarly, tumors adjacent to the psoas muscle often lack sufficient contrast for segmentation on CT and models may be augmented by MRI [25,26]. MR-assisted CT segmentation can be performed by simply referencing the MRI during the segmentation process or by super-imposing the MR images over the CT scan in a semi-transparent fashion. Therefore, ***even if a CT is used for 3-D modeling, a corresponding MRI should be obtained to facilitate manual segmentation in select cases (Evidence, 2.0 moderate).***

**Figure 5:**
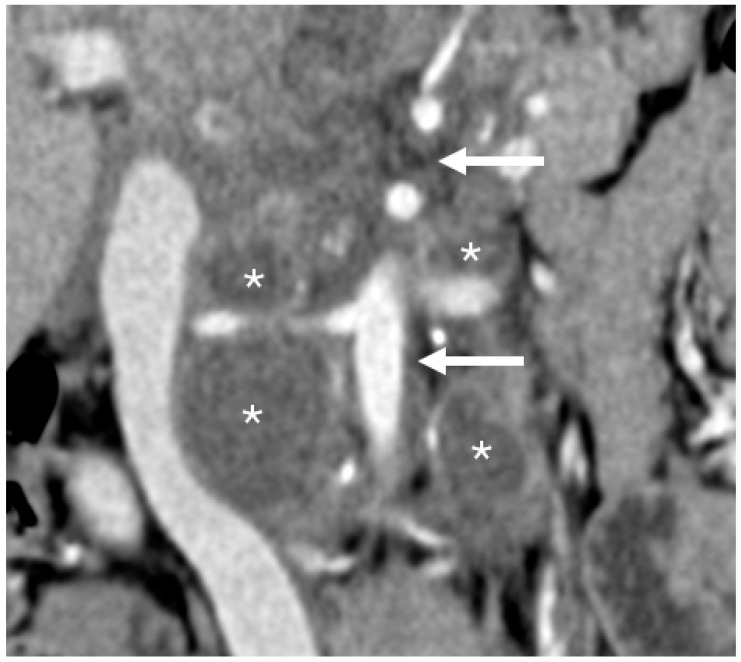
Coronal post-contrast CT image of a 5-year-old girl with neuroblastoma showing multiple retroperitoneal nodules (asterisks). In addition, there are areas of increased attenuation of the fat surrounding the aorta and its branches (arrows), without discrete margins, calcification, or mass effect. While these abnormalities may reflect post-chemotherapy changes rather than tumor, they should still be modeled as they can pose challenges to resection.

Following chemotherapy, but before surgery, tumors often shrink considerably in size, leaving behind a wake of abnormal retroperitoneal soft tissue. This post-chemotherapy retroperitoneal fat stranding can be defined on CT as abnormal attenuation of the retroperitoneal fat without calcification, rounded margins, or mass effect (Figure 5). ***These abnormal areas may represent scarring versus tumor and should be segmented separately from clearly defined tumors by imaging (Evidence, 2.25 moderate).*** Again, review of MRI, I-123 MIBG SPECT CT or PET/CT imaging, along with co-registered images may be helpful.

#### 3.5.2. Display

Once segmented, the models can be rendered to look photorealistic with a sense of depth and dimensionality. This is typically accomplished using simulated lighting models. While realistic, these 3-D-rendered virtual models on 2-D displays are still plagued by a fixed perspective and depth ambiguity [27,28]. This issue can be avoided by rotating the models as they are viewed. Two additional techniques are needed to overcome these limitations of fixed perspective and depth ambiguity: stereoscopy and parallax. Stereoscopy is the phenomenon whereby two images obtained from slightly different perspectives, when viewed by each eye and processed by the brain, produce a sense of depth and solidity. Parallax describes the phenomenon whereby the relative positions of objects appear to change as one moves around a scene [28]. Modern holographic, spatial, and virtual/augmented reality headsets incorporate parallax and stereoscopy to provide needed depth cues to allow the user to discern the anatomy being displayed without manipulation or rotation. The benefit of 3-D experiences to pre-operative planning is intuitive but lacks the tactile feedback provided by the 3-D printed models and needs further investigation. ***Nevertheless, parall*****ax *****and stereoscopic effects appear to improve understanding and should be incorporated when feasible (Evidence, 2.25 moderate) (Table 2)***.

**Figure 6:**
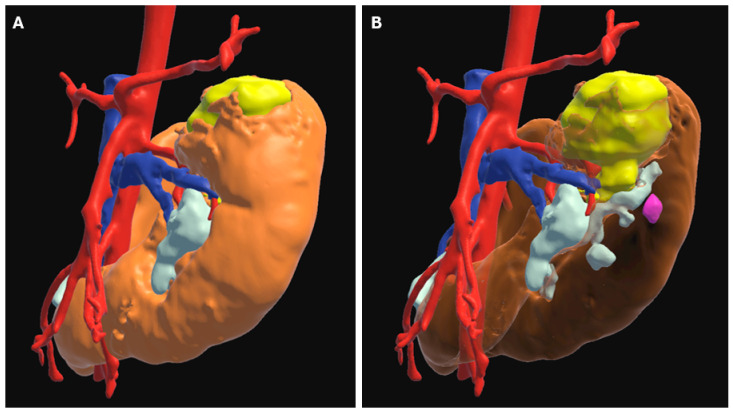
Three-dimensional model of a horseshoe kidney (orange) with a left superior pole tumor (yellow). (**A**) The kidney is rendered opaque which allows for lighting and shadows to convey the external contour of the kidney but obscures the tumor from view. (**B**) The kidney is rendered transparently, allowing visualization of the tumor within, but at the expense of losing monocular cues of shading to convey renal anatomy. Notice the tumor nodule shown in purple. While visible through the transparent kidney, its position relative to the kidney is uncertain. This limitation can be overcome by the use of rotatable images or stereoscopic effects, which provides a binocular cue of depth independent from lighting and shading.

Three-dimensional visualization and computer-aided design (CAD) software allows segmented objects to be made transparent, with only the edges visible. This can improve the 3-D understanding of complex structures. Transparent rendering and color coding is a helpful technique allowing for visualization of spatial relationships between two objects where, in real life, one would block visualization of the other. This facilitates visualization of vital structures encased by tumor, tumor within or invading organs, or tumors obscured by overlying normal structures. When utilizing transparent rendering, traditional lighting cues, such as object occlusion and shadows, are less effective at conveying depth (Figure 6). This limitation can be overcome using the principles of parallax and stereoscopy. The parallax effect can be simulated on a 2-D screen with a model which rotates with the scroll of the computer mouse; however, this may not be easily translated to the operating room environment. ***We recommend rotatable 3-D models on a 2-D display when performing transparent renderings (Evidence, 1.92 strong).***

However, rotating a 3-D rendering with a computer mouse does not take advantage of proprioceptive feedback which can improve spatial perception [28]. Alternatively, both parallax and stereoscopic effects are built into 3-D displays in the form of free-standing autostereoscopic or head-mounted displays. In both cases, the parallax effect is tied to head/body movement. In addition, the stereoscopic effect provides depth information without movement, which can be advantageous in an operating room setting, where mobility might be limited. ***Therefore, we recommend that the use of 3-D displays/headsets incorporating parallax and stereoscopic effects should be considered when rendering structures transparently (Evidence, 2.17 moderate).***

Image-guided surgical navigation of pediatric soft-tissue abdominal surgery poses unique challenges. The tissues involved are non-rigid and deformable, making correlation with pre-operative imaging difficult. Further, the soft-tissue structures do not contain fixed relationships to rigid structures, such as the spine, as the position and orientation of the spine relative to organs would likely differ between the CT and operating room tables. These factors make it difficult to accurately superimpose 3-D models onto the patient using augmented reality techniques. These are not insurmountable challenges, however, as translational, rotational, and deformational transformations can be applied in real time to the pre-operative models to match what is being viewed intra-operatively. ***However, this represents cutting-edge technology still under development for which we cannot make any recommendations at this time.***

## 4. Discussion

Innovations in 3-D visualization have several potential benefits to improving pediatric surgical oncology care in areas of staging, pre-operative planning, intra-operative assessment, and education for both trainees and families. As we apply these methodologies in clinical practice, standardization and quality assurance are critical to assess the true value of 3-D imaging technologies and optimize their use for pediatric patients. These guidelines propose minimum standards for image acquisition and 3-D model creation/visualization for pediatric solid tumors based on the literature and expertise with an overall high level of agreement amongst a multidisciplinary team.

Consideration for the purpose of the model will dictate the degree of accuracy required and impact decisions regarding segmentation and model viewing. When used for pre-operative planning, high-fidelity models are essential. However, when used for education purposes, less anatomic precision is likely needed. Patient selection will be crucial to guide imaging needs to ensure that the 3-D renderings support intended goals. Multidisciplinary discussion for each case is essential to ensure that acquisition protocols and segmentation processes will adequately address the clinical need and to honestly evaluate model quality and utility. While pediatric expertise from radiology and surgery is essential to select appropriate cases, identify the goal of modeling, and develop image acquisition protocols, engineering teams have a wide range of expertise and skills that likely vary by institution. Collaborative efforts to ensure quality standards are met will be critical as we advance and expand this technology into the forefront of pediatric surgical oncology.

The process to improve image quality and automate segmentation is already being catalyzed by artificial intelligence (AI), though the potential intersection of AI and 3-D modeling has yet to be fully elucidated. AI is currently being employed to improve image quality and to facilitate automated segmentation. This will lead to improved modeling and potentially lower radiation doses. Other possible implications of AI may include adaptive selection of printing materials for accurate texturing and tactile feedback, as well as expediting general manufacturing workflows to improve model throughput. The culmination of these technologies will lead to optimized modeling results while potentially lowering radiation doses for children.

Another advancement on the horizon is the photon-counting detector CT, which utilizes specialized detectors that directly convert incoming photons into electrical signal, bypassing the crystal fluorescence step utilized by traditional detectors [29,30]. The lack of crystal fluorescence allows detectors to be smaller (0.2 mm) thereby increasing the spatial resolution. These detectors are much more sensitive to incoming photons and can even detect the energy level of incoming photons, allowing for greater contrast resolution with decreased noise. The innate capabilities of this scanner will undoubtedly improve the quality of 3D models created.

Three-dimensional modeling for pre-operative planning in pediatric oncologic surgery exhibits tremendous utility and future potential. The increasingly available hardware and software required for 3-D modeling is reducing barriers to utilization. However, a lack of understanding of imaging requirements and processing currently hinders its acceptance as standard of care. The guidelines provided in this article reflect the minimum level of image quality needed to perform accurate modeling within the bounds of modern technology. While the recommendations are based upon the current landscape of available scanner technologies, rapidly evolving hardware and software solutions necessitate continual re-evaluation of standard of care imaging guidelines. Finally, guidelines must be tailored to the intent of the case at hand, as models for educating patients and families may not require the same fidelity as those being used for surgical planning and navigation.

## 5. Conclusions

Overall, the strength of recommendations for the proposed guidelines was high with most guidelines receiving very strong or strong recommendations. Guidelines pertaining to image acquisition were particularly well agreed upon, suggesting that incorporating these standards into image acquisition protocols may improve the quality of 3-D models for pediatric solid tumors and help expand the use of 3-D models across institutions by sharing institutional knowledge. Guidelines centered on viewing techniques were more variable, receiving moderate strength of recommendation, likely impacted by the individual expertise and familiarity with 3-D models at individual institutions and by surgeon preference. As the technology itself rapidly develops and familiarity with these techniques grows, preferences will likely evolve over time.

## Figures and Tables

**Table 1:** Image acquisition recommendations.

Recommendation	Evidence
Spatial resolution	
Imaging obtained for the purposes of 3-D modeling should be as thin as possible with isotropic voxels	Grade D, 1.33 very strong
MR sequences obtained for the purposes of 3D modeling should be as thin as possible without gaps between slices	Grade D, 1.17 very strong
Contrast	
Determining the structures of interest prior to the protocoling of the exam is important, as this may dictate the timing of contrast administration	Grade D, 1.25 very strong
When both vascular and ureteral opacification are desired, a multi-phase or split-injection technique should be used	Grade D, 1.42 very strong
MR-based 3D modeling of vascular structures remains a viable alternative to CT, depending on patient-specific factors	Grade D, 1.83 strong
Noise	
When acquiring images for 3D modeling, a low noise index should be used, though this will vary based on specific scanner hardware and software capabilities	Grade D, 1.58 strong

**Table 2:** Image post-processing recommendations.

Recommendation	Evidence
Segmentation	
Segmentations should be reviewed by a radiologist with pediatric imaging expertise	Grade D, 1.42 very strong
If a CT is used for 3-D modeling, a corresponding MRI should be obtained to facilitate manual segmentation in cases where tumor margins are indistinct on CT	Grade D, 2.0 moderate
Equivocal areas on imaging which may represent post-treatment change or tumor should be segmented separately from clearly defined tumor by imaging	Grade D, 2.25 moderate
Display	
Parallax and stereoscopic effects appear to improve understanding and should be incorporated when feasible	Grade D, 2.25 moderate
Rotatable 3-D models on a 2-D display should be utilized when performing transparent renderings	Grade D, 1.92 strong
3-D displays/headsets incorporating parallax and stereoscopic effects should be considered when rendering structures transparently	Grade D, 2.17 moderate

**Table 3:** CT and MRI for surgical planning.

Modality	Advantages and Disadvantages
CT	Advantages High spatial resolution Can obviate effects of motion with fast scans or adequate breath-holds Surgeons find it more comfortable to view Disadvantages Poor soft tissue contrast for some tumors Use of ionizing radiation Occasional need for sedation
MRI	Advantages Ability to obtain imaging at multiple timepoints following injection. Excellent soft tissue contrast Disadvantages Limited spatial resolution Motion artifact Frequent need for sedation

**Table 4:** Post-contrast acquisition timing tips for CT.

Vascular Phase *	Tips
Arterial	Can use bolus-tracking in the aorta to ensure proper timing. Newer scanners may not require high injection rates if sufficient SNR can be achieved.
Systemic Venous	Suprarenal IVC Often opacifies early due to inflow from renal veins. Infrarenal IVC Often difficult to opacify due to short injection time of weight-based contrast. In older/larger children, the increased contrast dose results in longer injection times, which may be sufficient for infrarenal IVC visualization. For younger/smaller children with pelvic masses, a lower-extremity injection can enhance visualization of the lower IVC and iliac veins.
Portal venous	Traditional portal venous timing applies. Often can be well visualized during late arterial phase.
Hepatic venous	Challenging to opacify in small children, similar to the infrarenal IVC. A split injection can help by allowing greater time for hepatic vein opacification while still maintaining good arterial opacification. For larger patients, a slow injection may allow enough time for hepatic vein opacification. Imaging at the end of injection without delay often provides sufficient arterial opacification.
Ureteral	15–20 min after injection. If using a split-injection technique, a small amount of contrast can be administered 15 min before scanning.

* MRI is more forgiving when it comes to the timing of imaging following contrast administration. However, the longer sequences result in respiratory motion artifact, particularly in children. Gadoxetate, a hepatobiliary agent, causes transient tachypnea and is also not as bright during the vascular phases. Therefore, traditional gadolinium agents should be used to visualize the hepatic veins on MRI.

## Data Availability

Data sharing is not applicable.
